# Gold and Tin in Saving Teeth

**Published:** 1890-07

**Authors:** C. R. Butler

**Affiliations:** Cleveland, Ohio.


ARTICLE III.

### GOLD AND TIN IN SAVING TEETH.

BY C. R. BUTLER, D. D. S., M. D., CLEVELAND, OHIO.

[Read before the Northern Ohio Dental Society, at Canton, Ohio, May, 1890.]

An old and interesting theme, yet ever new. First, because it has value; and then it must be ever new to the army of recruits that is added to the profession annually. And how shall they be able to take up improved modes and means of practice, unless these things are set plainly before them, as having passed the ordeal of time test, which is the prover of the faithful as well as the unfaithful efforts?

This subject—the combination of the two metals, in the same fillings—has claimed considerable attention, more especially the past few years.

The chemico-galvano electric feature of the presence of the metals in the teeth, the writer will not attempt to discuss at this time, that feature of the object having been brought before the profession by teachers of dental chemistry.

But we will take it up from a practical standpoint, and advantages observed after years of testing.

If some sixteen pages of arguement in one of the leading journals can be given to prove the great value of gutta-percha as an adjunct in dentistry, the writer will venture a few suggestions on the use of tin as a permanent filling in teeth.

How shall this be accomplished, for it is said that it wears out, no matter how well it is put in? Let us see whether this be true:

We will take, for example, proximal cavities in molars, or bicuspids, decay up to or under the free margin of the gum. The dam being adjusted, the preparation of cavity should be made with as much care as for gold, with angles as square as practicable, with slightest possible bevel of side margins of cavity, whether it be a deep or shallow cavity, so there shall be no feather-edge lap of metal on border of cavity when the filling is finished. Presupposing that working room has been secured, if possible would place a wedge between the teeth by the gum to hold them steady, then slipping a piece of steel down by lower margin of cavity, (the writer prefers an adjustable, rather than the closed band matrix.) We are now ready to commence the impactment of the filling, whether foil or fibrous tin be used in mat, strip, or pellet, by the operator, the all-important item being to have the metal packed into a solid mass; if this be done, close impingement upon the wall and border of the cavity will be secured.

It may not be out of place to mention the kind of instruments and force employed by the writer; the ordinary foot shape serrated points, (same instruments shown), with hand force in most cases; the hand mallet is also a good adjunct.

Having filled one-fourth or half the bulk of the cavity with tin, cut shallow retaining pits and grooves in the strong part of the tooth, and finish out the filling with non-cohesive or cohesive gold; it is not at all safe to depend upon the gold being driven into the tin alone for anchorage, to stand the force of mastication. But if the plan suggested be faithfully carried out, good safe contour fillings can be made.

One great cause of failure in the use of tin filling is, the operator does not start out with the idea that it is anything but an inferior material, and that fillings made with it are only temporary. But he who is fully imbued with the idea that the first and last object in filling teeth at all is to *save* them, and uses tin, *a la* Corydon Palmer, (who gave the writer instruction and hints many years ago, of the possibilities of tin,) will find that his works do follow for many years, not to condemn, but to encourage to be more and more faithful.

To all young subjects that require operations, the above mode of treatment of some cavities, proximate and buccal, is to be commended, for it is conceded by the best of operators that children's teeth are better saved with tin than with gold.

Tin has a fiber as well as gold, and should be cut with the fiber. No. 10 foil I prefer, taking a sheet in the hands with a to and fro movement, crimping it both ways, giving it a working property when cut into strips lengthwise of fiber, and rolled into soft cylinders of the size and length desired.

Do not think of applying to some manufacturer for made-up tin, but learn to prepare it for the case in hand. There is too much dependence on these "made-up" articles; they are often a misfit.

Some may decry this system of filling because it may be abused by the man who puts in more tin than gold, and represents to his patients that he is giving them gold operations. But that has the same degree of honesty that there is in representing that they are giving them first class gold fillings, that fail to give the comfort and service that should be had from plastics.

This mode of operating is neither cheap nor does it admit of an inferiority of skill in the technique. The plea that is made by a recent writer against contour and in favor of face fillings, that time and tax upon patient and operator are greatly lessened, has but little force in cases where a contour filling is demanded. And the man who attempts to fill all teeth in the same manner, or carry out some pet system, has failed to comprehend the factors that have so much to do in making operations upon the teeth at all necessary, and is scarcely worthy the name of dentist in this active age of careful observation.

If we are members of a "learned" profession, the laity have a right to expect better services than was obtainable by their ancestors. We often speak with pride of the wonderful advances made within the past half or quarter of a century in the art and science of dentistry, but the query often comes to me, are we doing as much to-day with all the boasted knowledge of the minute structural make-up of the teeth, and the almost endless fine adjuncts in the operative department to prevent and arrest decay of the teeth, as the men who had to depend so largely upon their individual effort for ways and means to meet the demands for dental work?

Whether it be a chemical or mechanical effect that the tin has upon the dentine, is still a mooted question. But we have observed that the higher the degree of vitality or predominance of animal matter in the tooth, the less it tolerates gold as a patch! So putting the tin where the major part of the cavity is dentine, then finishing out the masticating surface where enamel is mostly the receiving surface of the gold, the teeth are better preserved than when all gold was used! In many cases a mere line of tin is used along the basal margin. I have never been in favor of folding or rolling tin and gold together, but use them in definite sections.

The mode and results as given here are from actual practice. After years of testing, whether it be considered orthodox to declare such a line of practice, matters but little. My aim has been and is to make operations that save. One more example and I close this article. In cases where decay has made considerable inroads upon the proximate surfaces of the incisors, the structure of teeth is such that I should regard it the best kind of practice to open the cavities from the lingual surface. Secure as good borers as possible, fill with tin with a lining of non-cohesive gold under the face or labial wall, to modify the blue appearance that the tin might give the teeth. The matrix may be used to advantage in most of these cases, giving nature a chance under such fillings to harden the teeth.

No claim or care for priority is sought to be developed in this mode of using metals, as here presented. All operations, in fact, are but temporary.

But after years of careful observation of the mode and means employed to reach this class of cavities by men of skill and integrity, with the results following, I do not hesitate to put myself on record in support of this mode of making permanent fillings with tin and gold.—*The Ohio Journal of Dental Science.*

